# Some Practical Remarks Anent the Proposed Texas State Tuberculosis Sanitarium

**Published:** 1908-12

**Authors:** C. H. Wilkinson

**Affiliations:** Galveston, Texas


(Communicated.)

### Some Practical Remarks Anent the Proposed Texas State Tuberculosis Sanitarium.

BY C. H. WILKINSON, M. D., GALVESTON, TEXAS.

*Editor Texas Medical Journal:*

I have read the proposed bill for the establishment and maintenance of a sanitarium for the treatment and support of tubercular cases originating in the State of Texas, and I am gratified to note that it comprehends the great subject involved in nearly all of its essentials.

This bill was introduced in our last Legislature and is printed in full in the August number of the *Texas State Journal of Medicine* for this year. But for the great volume of business that accumulated on the hands of our legislators at that session it, no doubt, would have gone through at the time and would have been today the law of the land. However, it is believed that our forthcoming lawmakers will be ready to continue the question as soon as they have settled down to work in January next, and that ere another year has passed the construction of the Texas State Tuberculosis Sanitarium will be under headway. There is no greater need in our State at present for the development of any enterprise than there is for the establishment of some such institution as the one contemplated in the bill.

Texas is today, and has been for many years past, the dumping ground for hordes of indigent consumptives from the North and Middle Western States, and it requires no far-sighted sanitarian to forecast the evil consequences to our State if this undesirable inflation is not promptly counteracted or provided for.

The danger in this instance is the infection of our great State with a disease as difficult to eradicate from our midst as Johnson grass is difficult to be removed from the field of the farmer.

States can become infected just as cities and individual families can, through the continual harboring of infectious diseases in their midst. And this remark is as applicable to tuberculosis as it is to yellow fever, smallpox or bubonic plague. If it is appropriate for the State to care for her deaf and dumb, her epileptics and her blind, as certainly it is, then it is even more appropriate that she should provide in some manner for her consumptives. In the first instance cited only the afflicted individual is provided for, while in the latter case not only is the helpless individual taken care of and given a chance to regain his health, but, what is of more importance to the State, communities, localities and even entire towns and cities are thereby protected from a dire infection which once established destroys their salubrity, carries off their citizens, and for years thereafter renders them unfit places for the habitation of man. Once, within the memory of the writer, San Antonio's fame as a salubrious place of residence was unequaled by that of any other city in the United States. But, through the resorting there of large numbers of consumptives, many sections of this beautiful city, the fairest of the South, became infected and consequently undesirable for habitation.

This blight upon a naturally healthy city will in time become obliterated through the removal of the offending cause, but unless strenuous efforts are employed in this direction, no change for the better may be expected for many years to come.

Now, what has befallen San Antonio can reasonably be expected to happen to other towns and cities in our State to their great commercial disparagement. Our State's first duty in this connection should be to spare her principal cities and other communities from the dangers of tubercular infection by barring out the consumptive hordes that are pouring into our territory from other places; and, secondly, she should give her tubercular citizens a fair and rational showing for their lives. This last desideratum can only be brought about by the proper segregation of such invalids in a well-built, well-located and properly managed sanitarium, such as is contemplated in the proposed legislative act referred to, and it is now up to the lawmakers of Texas to establish such a sanitarium for our consumptives.

Glancing over the bill, as it appears in the Texas journal referred to, several features present themselves deserving of some special consideration.

The territory designated for the location of this institution is a vast area of healthy country,—all that section lying between latitudes 29 and 32 and west of longitude 98 in the State of Texas,—and, no doubt, in this broad area many ideal places might be found where a sanitarium could be eligibly located. But it must be borne in mind that many essentials are requisite besides salubrious atmosphere for the successful maintenance of a tuberculosis sanitarium, and chief among such essentials is the *accessibility* of such an institution.

Sanitariums should never be located at remote distances from cities or important trade centers, if it is practical to have them nearer, for many business reasons, and, if possible, they should always be located on some railroad. Consumptive cases require an occasional glimpse of civilization, and under proper restrictions they should be allowed at times to have it.

Again, to haul such cases over rugged country roads, in ordinary vehicles, for any great distance is expensive and inconvenient. Besides, remoteness from busy marts makes it difficult to obtain domestic help and other necessities so often needed in all institutions for the care of sick.

Ennui and loneliness act as depressants to tubercular cases, and it is important that they should be located where they can from time to time be allowed the opportunity of enjoying visions of healthy, animated life.

Nor should such institutions be established in or near a malarial neighborhood. A stream of water should, if possible, flow through the grounds of such a sanitarium, as is suggested in the proposed bill, but such a stream should be one of clear and running water, and not composed of sluggish pools, the natural breeding place of miasmatic germs.

Natural shade is likewise an important factor to be considered in this connection. It is essential to the comfort and well-being of the invalid, and the verdure it affords is pleasing to the eye and stimulating to the system.

As for the grounds connected with a tubercular sanitarium it is not necessary to have them very extensive. It is a mistake to imagine that a consumptive can perform much manual labor. Very slight exercise will bring on fever and aggravate his disease. When such cases are able to undertake efforts of this kind, they are no longer suitable inmates for a charitable institution, but might well be discharged therefrom and allowed to hustle for themselves.

As manager of Camp Reliance, at Comfort, Texas, for over two years I learned the truth of these assertions, and often I was forced to curtail the evening exercise of my patients for the reasons stated above. In an article I wrote upon this subject, and which was published in the Texas Medical Journal for April, 1907, I emphasized these facts, and called attention to many other features in connection with the location of a tuberculosis sanitarium.

Great care, therefore, should be exercised in locating any sanitarium for the treatment of tubercular cases, and the essentials which I have mentioned should ever be observed in order to insure the success of such an institution.

As to the *construction* of the buildings to be used: This matter should receive close and scientific attention. While the most salubrious district in the State might be selected for the location of a tubercular sanitarium, yet should the buildings to be used for such a purpose be improperly constructed they would prove practically useless, and the State's good intentions would be entirely subverted.

There is one element to be sought after in this connection without which few, if any, tubercular cases ever will be cured. That element is pure, fresh air in continuous quantities.

Modern researches prove that fresh air is indispensable for the cure of such cases, and its absence is the cause of the vast majority of deaths among consumptives.

An institution that does not provide for fresh air all the time day and night, summer and winter, is not constructed properly for the treatment of consumptives. Cases should be divided into three classes according to the stage of their disease and a separate building should be assigned to each of these three classes.

Such, then, are the main features that should be observed in the location and construction of a tuberculosis sanitarium. With all of these observed, such an institution will prove of the highest value to the State. With any of them omitted unsatisfactory results are sure to be experienced.

Reverting to other features of the proposed act, the writer ventures to offer some amendments towards perfecting this most excellent bill.

In Section 2 it is suggested "that said institution shall be known as the 'State Tuberculosis Sanitarium.' "

I would suggest that the qualifying adjective, "Texas," be appended so as to make the name of this institution read the "Texas State Tuberculosis Sanitarium." The suggested prefix is one that every loyal Texan is proud of; it is our trade-mark, as it were, and could never be appropriated by any other State.

Section 12 reads: "All persons afflicted with tuberculosis who shall have been bona fide residents of this State for two years next preceding the filing of their application with the county judge, as hereinafter provided, shall be admitted to said sanitarium under the provisions of this act."

Bearing in mind the motives that undoubtedly actuated the preparation of this bill, towit, the freeing of our towns and cities from the dangers of infection as well as affording a proper asylum for all tubercular cases, it becomes apparent that if this section be enforced only those who had been residents of Texas two years or longer would be eligible for admission into said sanitarium.

It is probable that many cases of tuberculosis develop in residents before they have been in the State even one year, and the question arises, what are we to do with such cases? To allow them to linger out their period of probation uncared for would be to endanger the lives of those who come in contact with them. In the opinion of the writer, consumptives should be handled somewhat like smallpox and other contagious cases. For the safety of themselves and those around them, they should be promptly separated from the unaffected and properly cared for.

The section referred to should be so worded and construed as as make all tubercular cases not properly cared for amenable to the law. That is, they should all be forced to enter the sanitarium unless otherwise properly provided for, no matter how short a time they had resided in the State.

Section 15 provides that no patient with a *transmissible disease* shall enter the sanitarium.

This restriction should be eliminated, since it does not aid the cause of sanitation, but, on the contrary, militates against it. Besides this, it is a well known fact that tuberculosis itself is one of the most transmissible diseases known.

Other sections of the bill might be modified somewhat, to their improvement, and probably they will be should the same ever come up before the Legislature for further consideration.

There is no provision made in the bill for funeral expenses, yet this is an important feature to have some understanding about beforehand, since in no diease in the world do as many funerals occur as in tuberculosis.

Taking it in its entirety, the proposed bill, less the exceptions noted, is an excellent and a praiseworthy one, since it earnestly aims at the deliverance of our State from a great and growing danger, and affords aid to a worthy class of citizens who otherwise would be unable to assist themselves.

---

THE IMMORTAL FIFTH (the San Antonio) District Medical Society honored itself by the election of Dr. Geo. H. Moody, of San Antonio, to the presidency at its regular semi-annual meeting in that city on November 20th, ult.

